# Antioxidant potential, cytotoxic activity and total phenolic content of *Alpinia pahangensis* rhizomes

**DOI:** 10.1186/1472-6882-13-243

**Published:** 2013-10-01

**Authors:** Chung-Weng Phang, Sri Nurestri Abd Malek, Halijah Ibrahim

**Affiliations:** 1 Institute of Biological Sciences, Faculty of Science, University of Malaya, Kuala Lumpur, 50603, Malaysia

**Keywords:** *Alpinia pahangensis*, Antioxidant, Phenolic content, Cytotoxic activity

## Abstract

**Background:**

*Alpinia pahangensis*, a wild ginger distributed in the lowlands of Pahang, Malaysia, is used by the locals to treat flatulence. In this study, the antioxidant and cytotoxic activities of the crude aqueous methanol and fractionated extracts of *Alpinia pahangensis* against five different cancer and one normal cell lines were investigated. The total phenolic content of each extract and its fractions were also quantified. This is the first report on the antioxidant and cytotoxic activities of *Alpinia pahangensis* extract.

**Methods:**

In the current study, the crude methanol and fractionated extract of the rhizomes of *Alpinia pahangensis* were investigated for their antioxidant activity using four different assays namely, the DPPH scavenging activity, superoxide anion scavenging, β-carotene bleaching and reducing power assays whilst their phenolic contents were measured by the Folin-Ciocalteu’s method.

*In vitro* neutral red cytotoxicity assay was employed to evaluate the cytotoxic activity against five different cancer cell lines, colon cancer (HCT 116 and HT-29), cervical cancer (Ca Ski), breast cancer (MCF7) and lung cancer (A549) cell lines, and one normal cell line (MRC-5). The extract that showed high cytotoxic activity was further investigated for its chemical constituents by GC-MS (gas chromatography–mass spectrometry) analysis.

**Results:**

The ethyl acetate fraction showed the strongest DPPH radical scavenging (0.35 ± 0.094 mg/ml) and SOD activities (51.77 ± 4.9%) whilst the methanol extract showed the highest reducing power and also the strongest antioxidant activity in the β-carotene bleaching assays in comparison to other fractions. The highest phenolic content was found in the ethyl acetate fraction, followed by the crude methanol extract, hexane and water fractions. The results showed a positive correlation between total phenolic content with DPPH radical scavenging capacities and SOD activities. The hexane fraction showed potent cytotoxic effect against KB, Ca Ski and HCT 116 cell lines with IC_50_ of 5.8 ± 0.1 and 9.1 ± 2.0 ug/ml, respectively. The major components of hexane fraction analysed by GC-MS analysis were mostly methyl esters.

**Conclusions:**

The current study suggests that the methanol extract and ethyl acetate fraction of *A. pahangensis* is a potential source of natural antioxidant for protective as well as prevention of life-threatening diseases. The hexane fraction of *A. pahangensis* may have the potential to be developed into therapeutic option for treating cancer.

## Background

*Alpinia* species, from the Zingiberaceae family have been extensively studied for their chemical and biological properties [1]. Based on the ethnobotanical studies, many species of this genus have been used in traditional medicine, and in the preparations and flavorings of food in many Asian countries. Among the *Alpinia* species, the rhizomes of *Alpinia galanga* have been widely used as spice and in the treatment of stomachic in China and Thailand; coughs, asthma, bronchitis, headache, inflammation, rheumatoid arthritis and colic in Malaysia [2,3]. One wild species, *Alpinia mutica* has been used to treat stomach upset by natives and it has also been reported to show good antioxidant and cytotoxic anticancer properties [4]. A few novel compounds isolated from the species of *Alpinia* had been found to have anti-cancer, anti-inflammatory, anti-spasmodic, anti-ulcerogenic, neuroprotective, analgesic, hepatoprotective and cardioprotective properties [5]. Due to its wide therapeutic values, it is of great interest to conduct more studies on the unexplored species from this genus which may possess medicinal properties, yet has not been fully studied. In this endeavour, *Alpinia pahangensis* was thus selected for investigation.

*Alpinia pahangensis*, a wild ginger, occcuring less common in the genus, is a perennial plant distributed in the lowlands of Pahang, Malaysia. The rhizomes of *A. pahangensis* have been used by tribal natives for relieving flatulence. However, there is a limited study on the biological activity of the *A. pahangensis*. A recent report by Awang et al. [6] showed that the essential oil extracted from the rhizomes and leaves of *Alpinia pahangensis* had good antimicrobial activity against *Staphylococcus aureus* strains and selected fungi.

Free radicals produced in our body due to aerobic respiration and substrate oxidation, can cause oxidative stress which may contribute to the development of several diseases including cancer, Alzheimer’s disease, aging, diabetes, Parkinson disease and atherosclerosis [7-13]. Overproduction of free radicals in our bodies may be increasing due to pollution and other external factors, and their removal by our antioxidant systems may be lower than before due to a number of factors related to our lifestyle among others. Oxidative stress causes serious damage to important cellular macromolecules such as protein and DNA. However, the production of free radicals can be balanced by antioxidant actions of endogenous enzymes as well as natural and synthetic antioxidants [14,15]. Antioxidants exert its action through several mechanisms including prevention of chain initiation, chelating of transition metal ion catalysts, decomposition of peroxidases, prevention of continued hydrogen abstraction and radical scavenging [16].

These deleterious effects of free radicals have drawn the attentions of scientists to the importance of antioxidants in prevention and treatment of diseases [17]. Thus, there has been increasing interest in finding natural diet-derived antioxidant to prevent oxidative damage [18,19]. Thus many studies have been carried out on natural sources to unravel the components which possess antioxidant properties and with low cytotoxicities [20]. Natural antioxidants are generally more desirable for consumption than the synthetic one such as butylated hydroxyanisole (BHA) which was reported to be carcinogenic to humans [21]. Recently, many studies have been carried out on the antioxidant properties of phenolic compounds which have aroused increasing interest in the isolation of such compounds present in the plants [22].

Cancer is a genetic disease, which is mainly driven by genetic instability, including changes in oncogenes and tumor suppressor genes which leads to the expression of abnormal proteins involved in the stimulation of cell proliferation and survival [23,24]. A large body of evidences have shown that free radicals have been implicated in the development of cancer in humans [25,26]. One example of the free radicals, is the hydroxyl radical which can cause genetic mutation by forming adduct with guanine to form hydroxylated bases of DNA (8 hydroxyl-2′-deoxyguanosine) causing transversions of GC (guanine-cytosine) to TA (thymine-adenine) [27,28]. Epidemiologic studies have also shown that cancer may be due to several factors such as exposure to environmental carcinogenic agents, lifestyle (tobacco and alcohol consumption), nutritional habit and infectious agents [29-32]. These factors can initiate and promote carcinogenesis which may progress to cancer.

To the best of our knowledge, there is no antioxidant and cytotoxic investigation on extracts of this species. Thus, this paper reports the antioxidant and cytotoxic activities of the crude aqueous methanol and fractionated extracts of the rhizomes of *Alpinia pahangensis* and to determine the phenolic content. This study also aims to correlate the phenolic content of the crude and fractionated extracts with its antioxidant properties. The active extract was further subjected to gas chromatography–mass spectrometry (GC-MS) analysis for identification of the components present in the extract.

## Methods

### Sample collection

The rhizomes of *Alpinia pahangensis* were collected from Pahang, Malaysia. This species was authenticated by Professor Dr Halijah Ibrahim, from Faculty of Science, University of Malaya and a voucher specimen (No. KLU 46177) deposited in the university herbarium.

### Reagents and chemicals

Butylated hydroxyanisole (BHA), ascorbic acid, Folin-Ciocalteu’s phenol reagent, β-carotene, linoleic acid, Tween 80, gallic acid and 2,2-diphenyl-1-picrylhydrazyl (DPPH), potassium ferricyanide were acquired from Sigma-Aldrich. Methanol, hexane, ethyl acetate and trichloroacetic acid were obtained from Merck. All solvents were purchased in analytical grade.

### Human cell line and culture medium

The cell lines were purchased from the American Tissue Culture Collection (ATCC, USA). The human cell lines used were nasopharyngeal epidermoid carcinoma cell line (KB), cervical carcinoma cell line (Ca Ski), colon adenocarcinoma cell line (HT-29), colon carcinoma cell line (HCT 116), lung adenocarcinoma epithelial cell line (A549), hormone-dependent breast carcinoma cell line (MCF7) and non-cancer human fibroblast cell line (MRC-5). The cells were propagated using the following growth media: RPMI (Sigma) for MCF7, Ca Ski, HT-29 cell lines, McCOY’s (Sigma) for HCT 116 cell line, and EMEM (Sigma) for MRC5 and KB cell lines, supplemented with 10% foetal bovine serum (PAA Lab, Austria), 100 μg/ml penicillin/streptomycin (PAA Lab, Austria) and 50 μg/ml of fungizone (PAA Lab, Austria). The cells were given new media every 2 to 3 days until 90% confluency. The viability of the cells was checked before and after treatment by the tryphan blue exclusion dye method. Frozen cell stocks were stored in liquid nitrogen (-196°C) prior to use.

### Extraction and fractionation

The dried, ground rhizomes of *Alpinia pahangensis* (200 g) were soaked in 80% aqueous methanol (3 L) for 3 days at room temperature. The solvent-containing extract was then filtered and the filtrate obtained was evaporated using a rotary evaporator at 40°C under vacuum to give the crude methanol extract (31.19 g, 15.60% based on the weight of dried, ground rhizomes). The crude methanol extract (31.19 g) was extracted with hexane (500 mL) and repeated three times (each time using 500 mL of hexane). The hexane-containing extracts were combined and concentrated *in vacuo* to give the hexane fraction (1.87 g, 6.00%). The hexane insoluble residue was further partitioned using ethyl acetate and water (500:500 mL) to give the ethyl acetate fraction (2.70 g, 8.66%) and the water fraction (24.43 g, 78.33%). The yield of crude methanol extract was calculated based on the weight of the dried, ground rhizomes whereas the yields of the fractions were calculated based on the weight of the crude methanol extract.

### Determination of total phenolic content

The total phenolic content was determined according to the Folin-Ciocalteu method as described by Phang et al [33]. The crude methanolic extract, hexane fraction, ethyl acetate fraction and positive control (BHA and ascorbic acid) were dissolved in methanol while water fraction was dissolved in distilled water. The total phenolic content (mg/g of plant extract) in the crude aqueous methanol extract and its fractions expressed in gallic acid equivalents (GAE). Mean values were calculated from three measurement.

### DPPH radical scavenging assay

The DPPH radical scavenging activity was determined using the method as described by Phang et al. [33]. An aliquot of extract of different concentrations were mixed with 0.8% of DPPH solution (0.02 mL) in methanol. Reaction mixtures were mixed well and incubated at room temperature for 30 minutes. Absorbance was read at 520 nm using spectrophotometer (UV-2450 Shimadzu). Methanol was used as blank and DPPH solution without addition of extract was used as control. BHA and ascorbic acid were used as standards. The percentage inhibition activity was calculated as [(A_0_ - A_1_)/A_0_] × 100, where A_0_ was the absorbance of the control, and A_1_ was the absorbance of the extract/standard. The IC_50_ value was determined by interpolation from non-linear regression of plot of percentage of inhibiton against the concentration of extracts, which is defined as the amount of extract needed to scavenge 50% of DPPH radicals.

### β-carotene bleaching assay

The antioxidant activity of the extract was determined according to the method of Phang et al. [33]. A reagent mixture was prepared containing of β-carotene (0.2 mg/ml in chloroform), linoleic acid (0.02 ml) and Tween 80 (0.2 ml). The reagent mixture was then transferred into a round bottom flask and the chloroform was removed using rotary evaporator. Oxygenated water (50 ml) was then added into the flask and shaken vigorously. Aliquots (5 ml) of the emulsion were transferred into test tubes containing 0.2 ml of extracts with different concentrations (4, 8, 16 and 20 mg/ml). After the emulsion was added into each test tube, the absorbance at zero time was measured immediately at 470 nm using a spectrophotometer (Genesys). The test tubes were then incubated at 50°C and the absorbance of each tube was measured again at time intervals of 20 minutes for 2 hours. The blank is the flask that is devoid of β-carotene whilst methanol is used as negative control. BHA was used as positive control.

The degradation rate of β-carotene (R) was calculated according to the equation below based on that described by Al-Saikhan et al. [34]:

$$R=\frac{1n\;\left(A_{0}/A_{t}\right)}{t}$$

where ln is natural logarithm, A_0_ is absorbance at time 0, A_t_ is absorbance at time t, and t is 20, 40, 60, 80, 100 or 120 minutes. The antioxidant activity (%) was calculated in terms of percentage inhibition relative to the control, using the equation below:

$$\text{Antioxidant}\;\text{activity}\;\left(\%\right)=\left(\frac{R_{{}_{\text{control}}-}R_{{}_{\text{sample}}}}{R_{{}_{\text{control}}}}\right)\times 100\%$$

### Reducing power assay

The reducing power was determined by the method of Murugan and lyer [35]. Different concentration of extracts (1, 0.5, 0.25, 0.125, 0.0625, 0.03125, 0.015625 mg/ml) dissolved in 1.0 mL of methanol, were mixed with 200 μL of 0.2 M phosphate buffer (pH 6.6) and 200 μL of 1% (w/v) solution of potassium ferricyanide. The mixture was incubated at 50°C for 30 minutes. Then, 200 μL of 10% (w/v) trichloroacetic acid solution was added after the mixture had cooled down. Aliquot of the upper layer (200 μL) was transferred to a 96 well plate and 20 μL of 0.1% (w/v) solution of ferric chloride was added. Absorbance of the reaction mixture was read at 620 nm in a plate reader (BioTek). Mean values from three measurement were taken. BHA and ascorbic acid were used as standards and the reaction mixture with methanol instead of the extract was used as (negative) control. The total reducing activity was determined by using formula:

$$\text{Total}\;\text{reducing}\;\text{activity}\;\left(\%\right)=1-\left(A_{c}/A_{t}\right)\times 100$$

Where:

A_c_ = Absorbance of control (reaction mixture with methanol instead of extract).

A_t_ = Absorbance with extracts/standards.

### Superoxide anion scavenging activity assay

The enzymatic antioxidant activity of the extract was determined using the SOD assay Kit-WST purchased from Sigma-Aldrich. The concentration of the extract/fractions and standards used was 5 mg/ml. This assay was done using 96 wells microtiter plate. Sample solution (20 μl) was added to sample well and blank 2 well, and 20 μl of ddH_2_O (doubled distilled water) was added to blank 1 and blank 3 wells. WST working solution (20 μl) was then added to each well and 20 μl of enzyme working solution was added to the sample well and the blank 1 well. The resultant mixtures were then mixed thoroughly. The plate was then incubated at 37°C for 20 min.

After incubation, the absorbance was read at 450 nm using an Elisa microplate reader. The superoxide anion scavenging activity was calculated according to the following equation:

$$\begin{array}{l} \text{SOD}\;\text{activity}\;\left(\text{inhibiton}\;\text{rate},\%\right)\\ \;=\left\{\left[\left(A_{\text{blank}1}–A_{\text{blank}3}\right)–\left(A_{\text{sample}}–A_{\text{blank}2}\right)\right]\right)\\ \;\left(/\left(A_{\text{blank}1}–A_{\text{blank}3}\right)\right\}\times 100 \end{array}$$

Where A_blank1_, A_blank2_, A_blank3_ and A_sample_ are absorbances of blank1, blank2, blank3, and sample wells. One unit of SOD activity was defined as the amount of enzyme having a 50% inhibitory effect on WST-1. The experiment was conducted in triplicates.

### *In vitro* neutral red cytotoxicity assay

The Neutral Red cytotoxicity assay used was based on the method described by Borenfreund and Puerner [36] with some modifications. Briefly, confluent cells were detached from the flask by incubating in 1 ml of 0.25% Trypsin-EDTA solution and were then seeded into sterile 96 wells microtiter plates (Nunc) at a density of 1 × 10^4^ cells per well. The cells were allowed to attach for 24 hours in a humidified 5% CO_2_ incubator at 37°C and maintained with growth medium. After 24 hours, the cells were treated with different concentration range of extracts (1, 10, 50, 100 ug/ml) for 72 hours. Doxorubicin was used as the positive control. The wells containing untreated cells were used as the negative control. At the end of the incubation period, the cells were incubated with media containing 50 μg/ml of Neutral Red for 3 hours. After 3 hours, the absorbance of dye eluted from viable cells was measured at 540 nm using a spectrophotometer Elisa plate reader (Molecular Devices EMax). The assay was carried out in triplicates. The concentration of extract which causes 50% inhibition or cell death is the 1C_50_. IC_50_ value for each extract was extrapolated from the graph plotted using the OD values obtained. The percentage of inhibition of each of the test samples was calculated according to the following formula:

$$\%\text{of}\;\text{inhibition}=\frac{{\text{OD}}_{\text{control}}-{\text{OD}}_{\text{sample}}}{{\text{OD}}_{\text{control}}}\times 100\%$$

Where OD _control_: Absorbance of negative control and OD _sample_: Absorbance of sample.

### Identification of the components

The GC-MS analysis was carried out using a Agilent Technologies 6980 N (United States) gas chromatography equipped with a 5979 Mass Selective Detector (70 eV direct inlet) and a HP-5 ms (5% phenylmethylsiloxane) capillary column (30 m × 25 mm × 0.25 mm film thickness) initially set at 100°C, then increased to 300°C and held for 10 minutes at ramp rate of 3°C per min using helium as the carrier gas at flow rate of 1 ml min^-1^. The total ion chromatogram obtained was autointegrated by Chemstation, and the components were identified by comparison with the accompanying mass spectral database (NIST 05 Mass Spectral Library).

### Statistical analysis

Data are expressed as mean ± SD of triplicates. Analysis of variance was used to determine any significant differences between groups using STATGRAPHICS Plus software (version 3.0, Statistical Graphics Corp., Princeton, NJ, USA). Statistical significance was accepted at p < 0.05. Duncan’s multiple range tests (DMRT) were used to determine the significant differences between groups.

## Results and discussion

### Amount of phenolic compounds in *Alpinia pahangensis* extract

Phenolic compounds are secondary metabolites that are derived from the pentose phosphate, shikimate and phenylpropanoid pathways in plants [37]. Phenolic compounds have been recognized to possess high antioxidant properties. The antioxidant activity of phenolic compounds is mainly due to their redox properties which allow them to act as radical scavengers, metal chelators, reducing agents, hydrogen donors, and singlet oxygen quenchers [38,39]. Thus, it is essential to evaluate the effect of the total phenolic content on the antioxidant activity of the extract and its fractions. Selection of solvents for extraction and fractionation is important in order to obtain desirable phenolic constituents. In general, aqueous alcohol (80% methanol and 70% ethanol) are the most preferred solvents to extract phenolic compounds from plants especially herbs [40,41].

Table 1 shows the yield of extracts/fractions and their respective total phenolic content. The highest amount of phenolic compounds (p < 0.05) was found in the ethyl acetate fraction which was 1.09 ± 0.11 mg of GAEs/g extract, followed by the crude methanol extract (0.75 ± 0.07 mg of GAEs/g extract), water fraction (0.61 ± 0.02 mg of GAEs/g extract) and hexane fraction (0.25 ± 0.03 mg of GAEs/g extract). This result suggested that extraction using polar solvents resulted in a higher content of phenolic compounds than those using solvent with low polarity.

**Table 1:** **Extraction yields and content of phenolic compounds in the crude and fractionated extracts of *Alpinia pahangensis***

**Extract/fractions**	**Weight of extracts (g)**	**Total phenolic content (mg/g)**
**Crude methanol**	31.19	0.75 ± 0.07^c^
**Hexane**	1.87	0.25 ± 0.03^a^
**Ethyl acetate**	2.70	1.09 ± 0.11^d^
**Water**	24.43	0.61 ± 0.02^b^

Values expressed are mean ± SD of triplicate measurements. Means with different letters (a-d) in the same column are significantly different (p < 0.05). mg/g: mg of gallic acid equivalents/g of extract or fractions.

### Determination of DPPH radical scavenging activity

This method has been widely used to evaluate the radical scavenging ability of the plant extracts as it is simple and highly sensitive. DPPH, a nitrogen-centered radical with a maximum absorption at 520 nm accepts an electron from an antioxidant which acts as a hydrogen donor. The scavenging activity of the extract was monitored based on the amount of DPPH radicals remaining in the test sample using a spectrophotometer. In our study, the highest scavenging effect was observed in the ethyl acetate fraction with an IC_50_ of 0.349 ± 0.009 mg/ml. This is followed by the crude methanol extract (0.579 ± 0.017 mg/ml), water fraction (0.999 ± 0.038 mg/ml) and hexane fraction (2.677 ± 0.094 mg/ml). However, BHA and ascorbic acid exhibited better scavenging ability than the ethyl acetate fraction. Table 2 shows the IC_50_ values of the crude extract and its fractions as compared to the standards, BHA and ascorbic acid.

**Table 2:** **Radical scavenging activity of the crude and fractionated extracts of *Alpinia pahangensis *against DPPH radical**

**Extracts and standards**	**IC**_**50**_**values (mg/ml)**
**Crude methanol**	0.579 ± 0.017 ^c^
**Hexane**	2.677 ± 0.094 ^e^
**Ethyl acetate**	0.349 ± 0.009 ^b^
**Water**	0.999 ± 0.038 ^d^
**Ascorbic acid**	0.015 ± 0.600 ^a^
**BHA**	0.013 ± 0.600 ^a^

IC_50_ values expressed are mean ± standard deviation of triplicate measurements. Means with different letters (a-e) in the same column are significantly different (p < 0.05). BHA and Ascorbic acid were used as standards.

### Determination of reducing power

**Figure 1:**
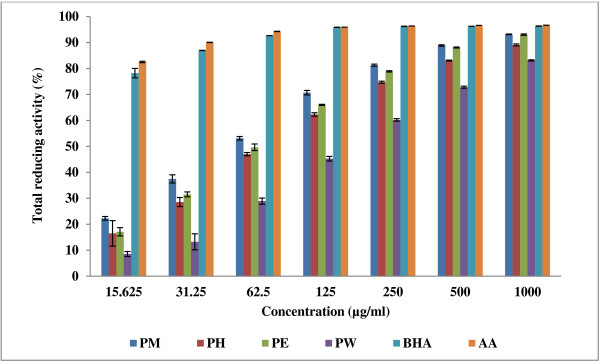
**The reducing power of the crude and fractionated extracts and the standards at various concentrations.** The concentrations of the crude and fractionated extracts were 1000, 500, 250, 125, 62.5, 31.25 and 15.625 μg/ml. Butylated hydroxyanisole and ascorbic acid were used as the standards. Values expressed are means ± standard deviation of triplicate measurements. PM., crude methanol extract, PH., hexane fraction, PE., ethyl acetate fraction, PW., water fraction, BHA., butylated hydroxyanisole, AA., ascorbic acid.

Figure 1 shows the reductive ability of the crude and fractionated extracts of the rhizomes of *A. pahangensis* in comparison to BHA and ascorbic acid. Reductive ability was measured by the reduction of ferricyanide complex/Fe^3+^ to the ferrous form (Fe^2+^) in the presence of antioxidant (reductant). The Fe^2+^ formation produce Perl’s Prussian blue and can be monitored at absorbance of 620 nm by a spectrophotometer. The reductive capability of the extracts and the standard compounds increased in the following order: water < hexane < ethyl acetate < methanol < BHA < ascorbic acid. The reducing power of the extract increased with the increase in concentration of the extract until it reaches a certain level and then become constant. Basically, reducing power is associated with the presence of reductones that break the free radical chain by donating a hydrogen atom [42].

### β-carotene linoleate model system

Bleaching of β-carotene of the crude and fractionated extracts was assessed by the β-carotene-linoleate method based on Cheung et al. In this system, linoleic acid undergoes oxidation and produce hydroperoxides at 50°C in the presence of oxygen. In the absence of antioxidants, the hydroperoxides formed upon abstraction of a hydrogen atom from one of its diallylic methylene groups reacts with unsaturated β-carotene molecules to form a stable radical. As a result, β-carotene becomes oxidized and loses its chromophore (orange color) in the system [43,44]. However, the presence of antioxidants can hinder the extent of β-carotene bleaching by neutralizing the linoleate-free radical and other free radicals formed in the system [45]. Therefore, the antioxidant activity was measured based on reduction of the orange color which was the amount of β-carotene present in the testing solution. The level of bleaching of color of a test solution was monitored at 470 nm. The antioxidant activities of the extracts varied significantly with different concentration of extracts (p < 0.05, Table 3). The antioxidant activity of the extract and its fractions and the standard compound increased in the following order: hexane fraction < water fraction < ethyl acetate fraction < methanol extract < BHA at every concentration. The antioxidant activity of the extract and its fractions increased with an increasing concentration of the extract as shown in Table 3. The crude methanol extract exhibited 81.21 ± 0.9% of antioxidant activity at 20 mg/ml, which was comparable to that of BHA at 4 mg/ml (81.51 ± 0.67%, Table 3).

**Table 3:** Antioxidant activity (%) of crude and fractionated extracts at various concentrations assayed by β-carotene bleaching assay

**Concentrations (mg/ml)**	**Antioxidant activity of crude methanol extract and its fractions**				
	**Methanol extract**	**Hexane fraction**	**Ethyl acetate fraction**	**Water fraction**	**BHA**
**4**	66.06 ±0.63^dw^	23.92 ±4.46^aw^	59.64 ± 1.44^cw^	50.03 ± 2.67^bw^	81.51 ± 0.67^ew^
**8**	74.73 ±1.84^cx^	42.17 ± 3.41^ax^	72.35 ± 2.30^cx^	57.54 ± 1.83^bx^	84.50 ± 0.21^dx^
**16**	77.84 ±0.41^cy^	52.62 ± 2.21^ay^	72.87 ± 1.73^bx^	71.74 ± 1.80^by^	89.54 ± 0.27^dy^
**20**	81.21 ±0.91^cz^	56.80 ± 2.70^ay^	74.51 ± 0.53^bx^	74.79 ± 1.56^by^	92.92 ± 0.23^dz^

Values expressed are mean ± standard deviation of triplicate measurements. For the same extract or standard with different concentrations, means in the same column with different letters (w-z) were significantly different (p < 0.05, ANOVA). For different extracts with the same concentration, means in the same row with different letters (a-e) were significantly different (p < 0.05, ANOVA). BHA was used as the standard.

### Superoxide anion scavenging activity

The superoxide anion scavenging ability of the extracts was determined using SOD assay kit-WST. Superoxide dismutase (SOD) is an enzymatic antioxidant that can scavenge superoxide anion radical (O_2_^-^) by catalyzing the dismutation of the superoxide anion into hydrogen peroxide and molecular oxygen. This assay is based on the measurement of superoxide dismutase inhibition activity. In this assay, the superoxide anion reduce WST-1 (2-(4-iodophenyl)-3-(4-nitrophenyl)-5-(2.4-disulfophenyl)-2H-tetrazolium) to generate the water-soluble formazan dye in the testing solution, which is measured spectrophotometrically at 450 nm. In the presence of an enzymatic antioxidant, the reduction of WST-1 can be inhibited by neutralizing O_2_^-^. Thus, the SOD activity can be quantified by measuring the decrease in the color development at 450 nm. The results in Table 4 show that the ethyl acetate fraction exhibited the highest superoxide anion scavenging ability with inhibition rate of 51.74 ± 4.9% among all extracts and fractions. This is followed by hexane fraction (32.21 ± 6.5%), methanol extract (29.32 ± 4.5%) and water fraction (18.06 ± 4.6%).

**Table 4:** Inhibiton rate (SOD activity) of the crude and fractionated extracts

**Extract/fractions**	**Inhibiton rate (%)**
**Methanol**	29.32 ± 4.5 ^b^
**Hexane**	32.21 ± 6.5 ^b^
**Ethyl acetate**	51.74 ± 4.9 ^c^
**Water**	18.06 ± 4.6 ^a^
**BHA**	70.19 ± 2.9 ^d^

Each value is expressed as mean ± standard deviation of triplicate measurements. Means with different letters (a-d) in the same column are significantly different (p < 0.05). Data are expressed as percentage of inhibition of superoxide radicals.

In summary, the crude and fractionated extracts of rhizomes of *Alpinia pahangensis* showed varying antioxidant properties in the entire *in vitro* antioxidant assays. The ethyl acetate fraction showed the greatest free radical quenching activity and superoxide anion scavenging activity associated with the highest amount of phenolic content. Thus, this shows that the phenolic content was positively correlated with DPPH radical scavenging activity and superoxide anion scavenging activity. As phenolic compounds have redox properties, this result is hardly surprising. The radical scavenging activity is usually related to the presence of hydroxyl substituents in aromatic rings, which contribute to their hydrogen donating activity [46]. Thus, the radical scavenging efficiency of the ethyl acetate extracts from *Alpinia pahangensis* might have been contributed by the phenolic constituents. However, the crude methanol extract possessed the strongest reducing activity against ferric ions and the highest antioxidant activity in the β-carotene bleaching assay. Lipid-soluble components are present in the crude methanol extract for the antioxidant activity shown in these two assays.

It was also observed that use of polar solvents like aqueous methanol and ethyl acetate resulted in the extraction of components showing good antioxidant activity whereas extracts obtained from non-polar solvents and water showed weak antioxidant activity. A wide variety of phenolic constituents has been reported in *Alpinia* species including flavonoids, tannins and some terpenoids. Thus, it is of interest to identify the compounds responsible for the antioxidant activity. Therefore, chemical isolation on the methanol or ethyl acetate fraction needs to be conducted to isolate the active components.

### *In vitro* neutral red cytotoxicity assay

*In vitro* cytotoxicity assays are widely used for drug delivery to evaluate the biological effects of chemicals on mammalian cells. Many currently available assays measure cytotoxicity based on alterations of plasma permeability and the leakage of components into the supernatant or the uptake of dyes, by viable cells [47]. In this study, quantification of number of viable cells in the culture was based on the ability of the viable cells to uptake neutral red which was incorporated into the lysosomes of the cells. Acidified ethanol solution was then used to extract the dye from the viable cells and the absorbance of the solubilized dye was then measured [48]. According to US NCI plant screening program, the extract that gave IC_50_ of 20 μg/ml or less is considered active whilst it is 4 μg/ml or less for pure compound [49,50].

Based on the result of the cytotoxic activity of the crude methanol and fractionated extracts (hexane, ethyl acetate and water) of *Alpinia pahangensis* (Table 5), the hexane fraction showed the highest cytotoxic activity with IC_50_ less than 20 μg/ml against KB, A549, Ca Ski, HCT 116 and HT-29 with the exception of MCF7. It also showed remarkable cytotoxic effect towards KB and HCT 116 with IC_50_ value of 5.8 ± 0.1 and 9.1 ± 2.0 μg/ml respectively. However, it also showed cytotoxic effect against the normal cell, MRC-5 with IC_50_ value of 17.3 ± 0.5 μg/ml. This was followed by the ethyl acetate fraction which showed strong cytotoxic activity against KB and HCT 16 with IC_50_ values of 10.2 ± 0.3 μg/ml and 19.9 ± 1.1 μg/ml respectively and moderate cytotoxic effect against other cell lines. However, the crude methanol and water fraction did not show cytotoxic effect against all cancer cell lines tested (IC_50_ more than 100 μg/ml). In this study, doxorubicin, a drug used for cancer chemotherapy as positive. Doxorubicin showed much higher cytotoxic activity against all the cancer cell lines tested in comparison to the hexane fraction and ethyl acetate fraction. Doxorubicin not only showed excellent cytotoxic effect against the cancer cell lines but it also showed strong cytotoxic effect against the normal cell line, MRC-5, with an IC_50_ value of 0.69 ± 0.05 ug/ml which is much higher than the hexane fraction.

**Table 5:** **IC**_**50**_**(μg/ml) values of crude and fractionated extracts of *Alpinia pahangensis *against selected cell lines**

**Extract/ fraction**	**Inhibition concentration, IC**_**50**_**(μg/ml)**						
	**KB**	**MCF7**	**A549**	**Ca Ski**	**HCT 116**	**HT-29**	**MRC-5**
**Methanol**	>100	>100	>100	>100	>100	>100	>100
**Hexane**	5.8 ± 0.1	22.0 ± 1.3	16.6 ± 0.2	18.4 ±0.2	9.1 ± 2.0	16.9 ± 0.5	17.3 ± 0.5
**Ethyl acetate**	10.2 ± 0.3	50.3 ± 0.3	25.9 ± 1.4	35.3 ± 2.0	19.9 ± 1.1	38.4 ± 0.4	44.0 ± 3.0
**Water**	>100	>100	>100	>100	>100	>100	>100
**Doxorubicin**	0.46 ± 0.02	0.088 ± 0.01	1.01 ± 0.02	0.31 ± 0.10	0.41 ± 0.07	0.56 ± 0.05	0.69 ± 0.05

Each value is expressed as mean ± standand deviation of triplicate measurements.

Data are expressed as IC_50_ in μg/ml which is the concentration of extract requires to inhibit cell growth by 50%).

In the GCMS analysis, the hexane fraction showed the presence of a major component, methyl palmitate (13.67%), and minor components such as methyl oleate (7.10%), methyl stearate (2.35%), 1,2-dimethyldecahydronaphthalene (3.05%), 1,5-dimethyldecahydronaphthalene (2.98%), 3,3,7,11-tetramethyl-tricyclo[6.3.0.0(2,4)] underc-8-ene (2.40%), 5-phenylundecane (0.82%) 6-phenyldodecane (0.87%) and 5-phenyldodecane (1.35%). Sri Nurestri et al.[51] reported that a mixture of methyl palmitate, methyl oleate and methyl stearate showed strong cytotoxic effect against Ca Ski, A549, as well as the normal cell line, MRC-5, with IC_50_ values less than 20 ug/ml. Methyl palmitate was also reported to exert cytotoxic effect on T-cell leukemia cell line (Molt-4) with an IC_50_ value of 2.28 ug/ml whilst methyl stearate showed cytotoxicity to acute promyeloblastic leukemia cell line (HL-60) and Molt-4 cell line with IC_50_ values of 3.08 and 4.65 μg/ml respectively [52]. In view of the above report, it is highly probable that the toxicity shown by the hexane fraction maybe partly due to the presence of methyl palmitate, methyl oleate and methyl stearate. The cytotoxic effect might be contributed by one or a combination of two or more of these components. Cytotoxic agents may cause necrosis in cells whereby cells lose membrane integrity leading to cell lysis or induce apoptosis cell death by activating an ordered series of biochemical events [53,54].

## Conclusions

This work describes for the first time the *in vitro* antioxidant and cytotoxic activity of the rhizomes of *Alpinia pahangensis*. The rhizomes showed good antioxidant capacity when evaluated against 5 antioxidant assays. The ethyl acetate fraction showed good DPPH radical scavenging and superoxide anion scavenging activities whilst the crude methanol extract possessed excellent reducing power ability almost comparable to that of the standards BHA and ascorbic acid, and good β-carotene bleaching activity. In contrast, the hexane extract showed good antiproliferative activity against KB and Ca Ski cell lines but weak antioxidant activity. It can thus be concluded that the rhizomes of *Alpinia pahangensis* have the potential to be used as chemopreventive and chemotherapeutic agent and consumption of these rhizomes may provide some health benefits. Further investigation on the underlying mechanism responsible for the biological activities should be attempted.

## Abbreviations

DPPH: 2,2-diphenyl-1-picrylhydrazyl; SOD: Superoxide dismutase; GC-MS: Gas chromatography–mass spectrometry; DNA: Deoxyribonucleic acid; GC: Guanine-cytosine; TA: Thymine-adenine; GAE: Gallic acid equivalents; BHA: Butylated hydroxyanisole; WST-1: 2-(4-iodophenyl)-3-(4-nitrophenyl)-5-(2,4-disulfophenyl)-2H-tetrazolium.

## Competing interests

The authors declare that they have no competing interests.

## Authors’ contributions

CWP was responsible for conducting the experiments, data analysis and interpretation, and preparing the manuscript. SNAM was responsible for providing the grants, conception of ideas, identification of components, and revising the manuscript. HI was responsible for providing grants, conception of ideas, collection and identification of plants, and revising the manuscript. All authors read and approved the final manuscript.

## Pre-publication history

The pre-publication history for this paper can be accessed here:

http://www.biomedcentral.com/1472-6882/13/243/prepub
